# Pure, shared, and coupling effects of climate change and sea level rise on the future distribution of *Spartina alterniflora* along the Chinese coast

**DOI:** 10.1002/ece3.5129

**Published:** 2019-04-16

**Authors:** Haibo Gong, Huiyu Liu, Fusheng Jiao, Zhenshan Lin, Xiaojuan Xu

**Affiliations:** ^1^ State Key Laboratory Cultivation Base of Geographical Environment Evolution (Jiangsu Province) Nanjing Normal University Nanjing China; ^2^ Key Laboratory of Virtual Geographic Environment, Ministry of Education Nanjing Normal University Nanjing China; ^3^ Jiangsu Center for Collaborative Innovation in Geographical Information Resource Development and Application Nanjing China; ^4^ College of Geography Science Nanjing Normal University Nanjing China

**Keywords:** climate change, ecological niche modelling, global sensitivity analysis, interaction, sea level rise, *Spartina alterniflora*

## Abstract

**Aim:**

Global change seriously threatens the salt marsh ecosystem, while it remains unclear how *S*. will respond to climate change and sea level rise. Here, we investigated interactions among variables and identified the impacts of climate change, sea level rise, and their interactions on the distribution of *Spartina alterniflora*.

**Location:**

Northern Chinese coast and Southern Chinese coast.

**Taxon:**

*Spartina alterniflora* Loisel.

**Methods:**

With global sensitivity analysis, we determined interactions among variables and their relative importance to the distribution of *S. alterniflora*. Integrating the Venn's four‐set diagram, we built ecological niche models under current and three future scenarios to identify pure, shared, and coupling effects of climate change and sea level rise on the distribution of *S. alterniflora*.

**Results:**

Mean diurnal range (Bio02) and Elevation were the two most critical variables controlling the distribution of *S. alterniflora *on the Chinese coast, and interactions among variables of the northern coast were much greater than that of the southern coast. Habitats change was mainly caused by pure effects of climate change, except habitats reduction on the southern coast. Pure effects of sea level rise were low, but it can influence habitats change through shared and coupling effects from complex interactions with climate change. Interactions of climate change and sea level rise can drive habitats change, and the changed habitats caused by shared and coupling effects were mainly distributed the areas near the landward side.

**Main conclusions:**

Our research suggests paying attention to interactions among variables when calculating the relative importance of explanatory variables. Identifying pure, shared, and coupling effects of climate change and sea level rise for the distribution of *S. alterniflora* will provide scientific references for assessing the risk of similar coastal species.

## 1 INTRODUCTION

It is now well established that the earth's climate is warming (Cazenave & Cozannet, 2014; Priest et al., 2010) and that the rate of sea level rise is accelerating, with a projection of global sea level rise of 0.75–1.90 m by next century (Team et al., 2014). Although previous studies have reported the impacts of projected sea level rise on salt marsh plant communities (Allen & Lendemer, 2016; Donnelly & Bertness, 2001; Kirwan et al., 2010; Valle et al., 2014), few investigators have examined the interactive effects of sea level rise and climate change for the salt marsh plants (Garner et al., 2015; Kirwan & Mudd, 2012). Their interactions and compounding effects may lead to reduced salt marsh sustainability (Charles & Dukes, 2009; Cherry, Mcknee, & Grace, 2009) and influenced the ability of salt marsh plant to survive (Kirwan et al., 2013). Kirwan (2012) also found that plants responses differed depending upon the elevation of the marsh relative to sea level under interactive effects. Recent studies showed that interactions may further change the composition of species assemblages and making important ecological processes at salt marshes uncertain in the future (Garner et al., 2015; Hanson et al., 2016). So far, there has been little discussion about habitats change caused by the pure effects of climate change, sea level rise, and the shared and coupling effects from their interactions. Quantifying these effects will help us better understand the effects of climate change and sea level rise on the coastal ecosystem, and further accurately assess the risk caused by them.

Coastal ecosystems are expected to be exposed to the increased risk of experiencing adverse consequences related to climate change and exacerbated by rising sea level (Nicholls et al.,^2007^; Valle et al., 2014), while it is still unclear how coastal ecosystems respond to them. It is possible that in coastal ecosystems, native species will decline due to their poor adaptability to these threats (Mendoza‐Gonzalez et al., 2013). However, invasive exotic species may take the opportunity to expand their habitats and stabilize their colonial status. So, understanding how invasive coastal species respond to climate change and sea level rise is becoming an urgent challenge (Brierley & Kingsford, 2009; Hoegh‐Guldberg & Bruno, 2010).

*Spartina alterniflora* Loisel, native to the Atlantic and Gulf coasts of North America (Wang et al., 2006), is a highly invasive species widely distributed along the Chinese coast (Yang et al., 2008). A large number of published studies have already revealed the physiological characteristics and expansion mechanisms of *S. alterniflora* based on laboratory work (Deng et al., 2006; Hu et al., 2015; Shi et al., 2007; Wang et al., 2015; Gu & Zhang, 2009; Zhao et al., 2007; Li et al., 2018). These studies demonstrated that the expansion of *S. alterniflora* is influenced by elevation, climate, soil salinity, inundation duration, pH, and many other variables could influence, but previous studies only focus on the single‐variable and ignore the interactive effects of multiple variables (Braun, Schindler, & Rihm, 2017; Daniel, Hubert, GertJan, & Wim, 2009). Although studies have recognized the importance of interactions, fewer researches have systematically identified interactions among variables (Liu et al., 2018). Moreover, as a salt marsh plant, the distribution of *S. alterniflora* is peculiarly prone to the impacts of coastal change. *S. alterniflora* is correlated with variations in sea level, and its productivity peaks at intermediate elevations within the intertidal zone (Kirwan et al., 2013). However, no research has surveyed the response of *S. alterniflora* to climate change, sea level rise, and their interactions. Through the maximum entropy model, we built ecological niche models to explore the response of *S. alterniflora* to climate change, sea level rise, and their interactions under three future scenarios (only considering climate change, only considering sea level rise and both considering climate change and sea level rise) on the northern and southern Chinese.

Specifically, the following issues will be addressed: (a) whether there are interactions among variables influencing the distribution of *S. alterniflora*, and how the role of the key variable varied according to different regions. (b) Identifying pure, shared, and coupling effects of climate change and sea level rise on the future distribution of *S. alterniflora* and assessing the relative importance of them on different regions. (c) Determining the spatial distribution of *S. alterniflora *caused by climate change, sea level rise, and their interactions.

## 2 MATERIALS AND METHODS

### 2.1 Study area

**Figure 1:**
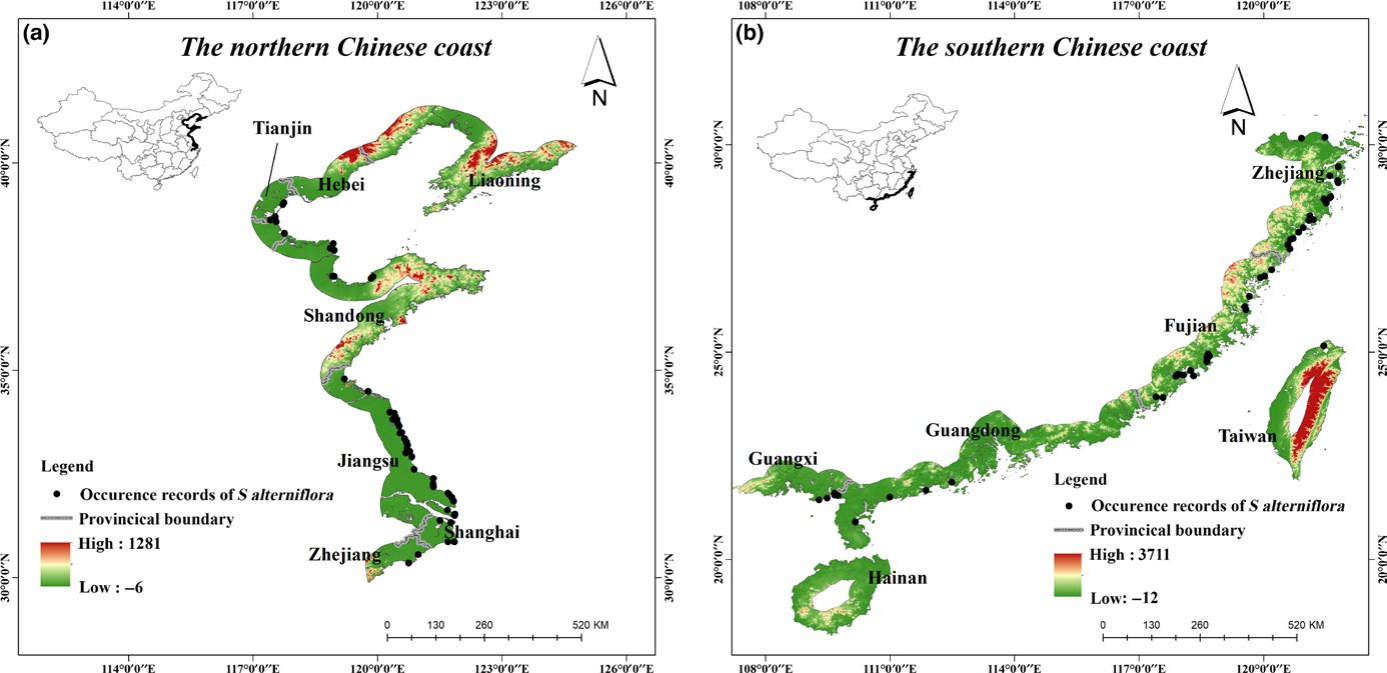
Study area. (a) the northern Chinese coast (b) the southern Chinese coast

The geographical extent of the study area was obtained by a 50‐km inland buffer of the shoreline of China including 14 Chinese provinces. According to different coast types and colonization characteristics of *S. alterniflora *(Gao et al., 2014), the study area was divided into two regions: northern Chinese coast and southern Chinese coast (Figure 1).

### 2.2 Data sources

Most of presence records of *S. alterniflora* on Chinese coast were obtained from published studies (An et al., 2007; Xie & Gao, 2009; Zhang et al., 2010; Zhang et al., 2008; Zhao et al., 2015; Zheng et al., 2018). The others were obtained from field sampling, and the Global Biodiversity Information Facility (available at http://data.gbif.org/). The presence records were resampled in ArcGIS 10.2 to ensure that there is only one observation within the 1° by 1° cell to avoid spatial autocorrelation and reduce sampling bias (Merckx et al., 2011), resulting in retention of 106 presence records. For the northern Chinese coast and southern Chinese coast, there were 56 presence records and 48 presence records, respectively.

We selected three factor types including climate, topography, and soil as environmental variables. A total of 19 bioclimatic variables were obtained from Worldclim (www.worldclim.com; Hijmans et al., 2005). Bioclimatic data include two groups, one of which is 19 bioclimatic under current conditions and the other is 19 bioclimatic variables of future climatic conditions (RCP 8.5: A scenario of comparatively high greenhouse gas emissions, Riahi et al., 2011). Considering the collinearity among bioclimatic variables may lead to overfitting, we used the following measures to reduce the number of variables. Firstly, using Spearman rank correlation coefficients, we eliminated bioclimatic variables with the highest and most significant correlation coefficients (***|*** *r* ***| ***> 0.8 and *p* < 0.001) (Supporting information Figure S1). Then, Boruta, a wrapper built around the random forest classification algorithm implemented in the R, was used to select variables according to the relative importance of bioclimatic variables (Supporting information Figure S2) (Kursa, Jankowski, & Rudnicki, 2010). Finally, six bioclimatic variables (Mean Diurnal Range, Isothermality, Mean Temperature of Wettest Quarter, Mean Temperature of Warmest Quarter, Precipitation of Wettest Quarter, Precipitation of Coldest Quarter) were determined as climatic variables. Topography variable was represented by elevation data at 30 arc‐second‐cell resolution was downloaded from IIASA (http://webarchive.iiasa.ac.at/Research/LUC/External-World-soil-database/HTML/). Furthermore, we obtained the global mean sea level when the sea level rises by 1 meter from CReSIS (https://www.cresis.ku.edu/content/research/maps). Seven soil variables (Soil electrical conductivity, Soil organic carbon, Soil pH, Percentage sand, Volume percentage gravel, Soil unit symbol, and Soil drainage class) were derived from the Harmonized World Soil Database (HWSD) version 1.2.1 with a spatial resolution of 1 km.

The spatial resolution of all variables was resampled into 1km to match those of the environmental variables (Supporting information Table S1 and S2) with the nearest‐neighbor approach in ArcGIS 10.2.

### 2.3 Ecological Niche Modeling based on MaxEnt

MaxEnt, one of the most popular machine algorithms, is designed for modeling the geographical distribution of species from the *n*‐dimensional environmental variables spaces with presence‐only data (Phillips et al., 2006). A 10‐fold cross‐validation procedure, which is preferable to penalty functions for assessing model generality, was implemented to replicate model runs and data partitions (Merow et al., 2013). It holds out 10% of the data as a testing set at each of 10 iterations, training the model on the remaining 90% of the data in each iteration. Other specified parameters and their setting are maximum number of background points = 10,000, Maximum iterations = 1,000, Convergence threshold = 0.00001, prevalence = 0.5.

### 2.4 Global sensitivity analysis based on FAST method

Sensitivity analysis (SA) is the study of how the uncertainty in the output of a model can be apportioned to different sources of uncertainty in the model input (Saltelli & Homma, 1992) and is usually divided into local sensitivity analysis and global sensitivity analysis. Compared to local sensitivity analysis, the range of variation of the parameters of the global sensitivity analysis can be expanded to the entire domain and interactions between parameters can be considered (Haaker & Verheijen, 2004). The Fourier amplitude sensitivity test (FAST) method, based on performing numerical calculations to obtain the expected value, is more efficient to calculate sensitivities than other variance‐based global sensitivity analysis methods (Dan, 2010; Mcrae et al., 1982; Saltelli & Bolado, 1998). The FAST method give first‐order sensitivity indices (*S_F_*) and total sensitivity indices (*S_T_*) using the terms in the Fourier decomposition of the model output. The *S_F_* measures the main effect contribution of each variable to the total output variance, and the *S_T_* accounts for the total contribution including main effects and interactions effects (Vanuytrecht et al., 2014). The difference between *S_T_* and *S_F_* which can assess the impacts of interactions among variables (Nossent, Elsen, & Bauwens, 2011).

In order to identify interactions among variables and their relative importance for the distribution of *S. alterniflora* on different regions, we performed the FAST sensitivity analysis with Simlab software version 2.2 (Joint Research Centre of the European Commission, 2011). The probability distribution functions were generated for all variables and significance levels were set at the 1% level. All fitted results passed the chi‐square test (*p* < 0.005) (Supporting information Table S1 and S2). The method of Fast (Saltelli et al., 1999) requires $N=(2M*W_{\max}+1)*m$ model simulations, where *M *is the interference factor (*M* = 4), $W_{\max}$ is the largest among the set of $W_{i}$ frequencies ($W_{\text{max}}$=416), *m* is the number of input factors (*m* = 15), and *N* is the total number of parameter sets and model executions. A total of 49,935 input parameter sets were generated using probability distribution functions on different regions with Simlab software version 2.2. These parameter sets were run in MaxEnt and then used for global sensitivity analysis in Simlab.

### 2.5 Model tuning and evaluation

Feature types combination (FC) and regularization multiplier (RM) are two important parameters that affect model complexity (Merow et al., 2013; Muscarella et al., 2015). ENMeval, an R package, was proved to be useful for tuning these two parameters (RM and FC) (Muscarella et al., 2015). Thus, corrected Akaike information criteria (AICc) value was used to estimate the model complexity in MaxEnt (Dan & Seifert, 2011).The smallest AICc was chosen for model simulation and was thought to can reduce model complexity relative to the default model.

The threshold‐independent and threshold‐dependent measures were used to evaluate model performance. Area under the curve (AUC) metric, a typical threshold‐independent measure, was utilized as a measure of model accuracy ( Lobo et al., 2008). Values of AUC generally range from 0.5 (equivalent to that due to chance) to 1.0 (perfect performance). Values > 0.9 are considered good, 0.7–0.9 are moderate, and <0.7 are poor (Fielding & Bell, 1997). The true skill statistic (TSS), a commonly used threshold‐dependent measure of model accuracy(Allouche, Tsoar, & Kadmon, 2006), is calculated as sensitivity + specificity −1. Values > 0.6 are considered good, 0.2–0.6 are fair to moderate, and < 0.2 are poor (Allouche et al., 2006).

### 2.6 Identifying pure, shared, and coupling effects of climate change and sea level rise on species distribution

**Figure 2:**
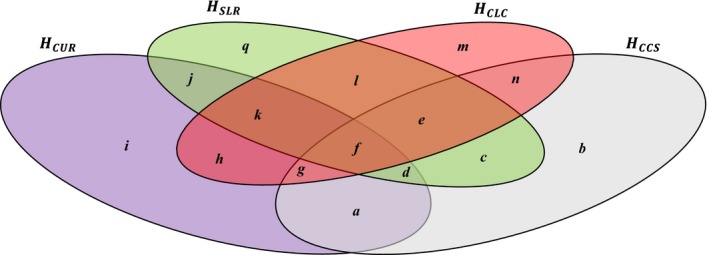
The Venn's four‐set diagram. Based on the current habitats and future habitats, we could find the changed habitats (*H*_changed_ = *b*+*c* + *e*+*n* + *i*+*j* + *h*+*k*) and unchanged habitats (*H*_unchanged_ = *a*+*d* + *f*+*g*). For the changed habitats, we divided it into the increased habitats ($H_{\text{changed}}^{+}=e+c+n+b$) and decreased habitats ($H_{\text{changed}}^{-}=i+j+h+k$). For the increased and decreased habitats, they could be spilt into 4 parts. We define *e *($H_{s\_ics}^{+}$) and *k *($H_{s\_ics}^{-}$) as habitats caused by the shared effects due to interactions of climate change and sea level rise, *c* ($H_{p\_slr}^{+}$) and *j* ($H_{p\_slr}^{-}$) as habitats caused by the pure effects of sea level rise, *n* ($H_{p\_clc}^{+}$) and *h* ($H_{p\_clc}^{-}$) as habitats caused by the pure effects of climate change, and *b* ($H_{c\_ics}^{+}$) and *i* ($H_{c\_ics}^{-}$) as habitats caused by the coupling effects due to interactions of climate change and sea level rise

To explore the impacts of climate change, sea level rise, and their interactions on the distribution of *S. alterniflora*, we designed three future scenarios using MaxEnt. The scenario of climate change (CLC), under which climatic variables changed but remaining variables kept constant, obtained habitats when only considering climate change. The scenario of sea level rise (SLR), under which elevation changed but remaining variables kept constant, obtained habitats when only considering sea level rise. The scenario of combining climate change and sea level rise (CCS), under which climatic and soil variables were changed, obtained habitats when considering both climate change and sea level rise. We modeled the current potential distribution of *S. alterniflora *along the northern and southern Chinese coast and projected them into future under three scenarios of CLC, SLR, and CCS. Given the uncertainty of the MaxEnt output (Hanberry & He, 2013; Swanson et al., 2013), three threshold rules, such as the maximum training sensitivity plus specificity cloglog threshold (MTSS), 10% training presence cloglog threshold (PTSS), and equal training sensitivity and specificity cloglog threshold (ETSS), were employed to calculate the weighted average threshold based on the results of the TSS evaluation. Then, we set the suitable habitats of *S. alterniflora *under current (CUR) and future scenarios (CLC, SLR, CCS) as respectively. The Venn's four‐set diagram, showing all possible logical relations between a finite collection of different sets (Henderson, 1963), was implemented to identify the impacts of climate change, sea level rise, and their interactions (Figure 2). Conceptually, four ellipses represented the current habitats ($H_{\text{CUR}}$) and three kinds of future habitats ($H_{\text{CLC}}$, $H_{\text{SLR}}$, and $H_{\text{CCS}}$) (Figure 2). Based on $H_{\text{CUR}}$ and $H_{\text{CCS}}$ *,* we defined the changed habitats ($H_{\text{changed}}$) and unchanged habitats($H_{\text{unchanged}}$):

(1) $$H_{\text{unchanged}}=H_{\text{CCS}}\cap H_{\text{CUR}}=a+d+f+g$$

(2) $$H_{\text{changed}}=H_{\text{CCS}}\cup H_{\text{CUR}}-H_{\text{unchanged}}=b+c+e+n+i+j+k+h$$

For the changed habitats of *S. alterniflora*, it was spilt into eight parts in Equation (2). For *c* and *j*, they belonged to the habitats when just considering sea level rise, so they were assumed as increased and decreased habitats caused by pure effects of sea level rise (*H_p_*___ *_slr_*). Similarly, *n* and *h* are increased and decreased habitats caused by pure effects of climate change (*H_p_*___ *_clc_*). *e *and *k* are increased and decreased habitats shared by *H*_CLC_ and *H*_SLR_, and we assumed *e* and *k* are caused by shared effects due to interactions of climate change and sea level rise (*H_p_*___ *_ics_*). For *b* and *i*, they did not belong to habitats when considering only climate change or sea level rise, so, we assumed them as increased and decreased habitats caused by coupling effects due to interactions of climate change and sea level rise (*H_c_*___ *_ics_*).

Specifically, the decreased suitable habitats ($H_{\text{changed}}^{-}$), the decreased habitats caused by shared effects ($H_{s\_ics}^{-}$), pure effects of climate change ($H_{p\_clc}^{-}$), pure effects of sea level rise ($H_{p\_slr}^{-}$), and coupling effects ($H_{c\_ics}^{-}$) can be expressed as the following:

(3) $$H_{\text{changed}}^{-}=H_{\text{CCS}}\cup H_{\text{CUR}}-H_{\text{CCS}}=i+j+k+h$$

(4) $$H_{s\_ics}^{-}=(H_{\text{CLC}}\cap H_{\text{CUR}})\cap \left(H_{\text{SLR}}\cap H_{\text{CUR}}\right)\cap H_{\text{changed}}^{-}=k$$

(5) $$H_{p\_clc}^{-}=\left(H_{\text{CLC}}\cap H_{\text{CUR}}\right)\cap H_{\text{changed}}^{-}-H_{s\_ics}^{-}=h$$

(6) $$H_{p\_slr}^{-}=\left(H_{\text{SLR}}\cap H_{\text{CUR}}\right)\cap H_{\text{changed}}^{-}-H_{s\_ics}^{-}=j$$

(7) $$H_{c\_ics}^{-}=H_{\text{changed}}^{-}-H_{p\_clc}^{-}-H_{p\_slr}^{-}-H_{s\_ics}^{-}=i$$

Similarly, the increased suitable habitats ($H_{\text{changed}}^{+}$), the increased habitats caused by shared effects ($H_{s\_ics}^{+}$), pure effects of climate change ($H_{p\_clc}^{+}$), pure effects of sea level rise ($H_{p\_slr}^{+}$), and coupling effects ($H_{c\_ics}^{+}$) can be expressed as the following:

(8) $$H_{\text{changed}}^{+}=H_{\text{CCS}}\cup H_{\text{CUR}}-H_{\text{CUR}}=b+c+e+n$$

(9) $$H_{s\_ics}^{+}=\left(H_{\text{CLC}}\cup H_{\text{CUR}}-H_{\text{CUR}}\right)\cap (H_{\text{SLR}}\cup H_{\text{CUR}}-H_{\text{CUR}})\cap H_{\text{changed}}^{+}=e$$

(10) $$H_{p\_clc}^{+}=\left(H_{\text{CLC}}\cup H_{\text{CUR}}-H_{\text{CUR}}\right)\cap H_{\text{changed}}^{+}-H_{s\_ics}^{+}=n$$

(11) $$H_{p\_slr}^{+}=\left(H_{\text{SLR}}\cup H_{\text{CUR}}-H_{\text{CUR}}\right)\cap H_{\text{changed}}^{+}-H_{s\_ics}^{+}=c$$

(12) $$H_{c\_ics}^{+}=H_{\text{changed}}^{+}-H_{p\_clc}^{+}-H_{p\_slr}^{+}-H_{s\_ics}^{+}=b$$

Finally, according to Equation ((3), (4), (5), (6), (7), (8), (9), (10), (11), (12)), we quantified pure, shared, and coupling effects of climate changes and sea level rise and identified their spatial distribution using spatial analysis tool in ArcGIS 10.2.

## 3 RESULTS

### 3.1 Model performance

**Figure 3:**
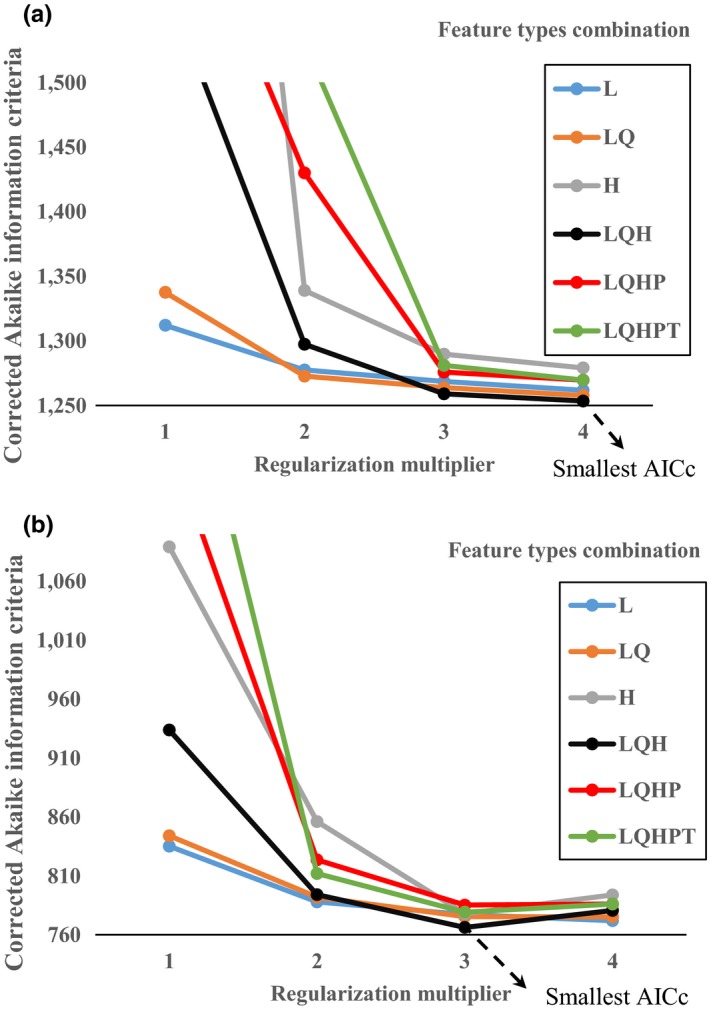
AICc and smallest AICc values of ecology niche models with different regularization multiplier and feature types combination. (a) the northern Chinese coast (b) the southern Chinese coast

To reduce model complexity and avoid overfitting, RM and FC with the smallest AICc values were chosen in MaxEnt as shown in Figure 3. RM = 3 and FC = “LQH” were chosen on the northern Chinese coast (Figure 3a). RM = 4 and FC = “LQH” were chosen on the southern Chinese coast (Figure 3b).

**Figure 4:**
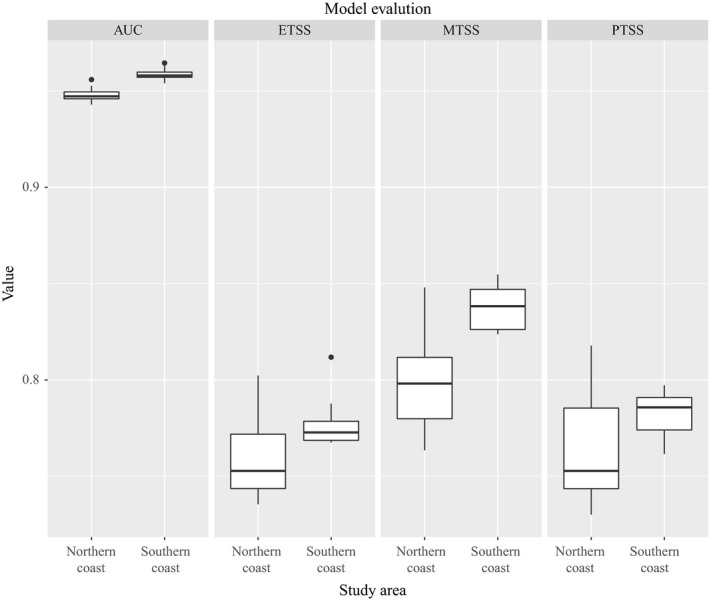
Model evaluation metrics for ecology niche models at the northern Chinese coast and southern Chinese coast using Area Under the Curve(AUC) and true skill statistic(TSS)

The results of model evaluation showed that the AUC values were all greater than 0.9 and the values of TSS (three threshold rules such as the maximum training sensitivity plus specificity cloglog threshold, 10% training presence cloglog threshold, and equal training sensitivity and specificity cloglog threshold) were greater than 0.7 in Figure 4. So all the models performed well.

### 3.2 Interactions among variables and their relative importance for the distribution of *S. alterniflora* on different regions

As shown in Table 1, on the northern Chinese coast, mean diurnal range (Bio02), as well as elevation, had much higher first‐order sensitivity indices (*S_F_*) and total sensitivity indices (*S_T_*) than the other variables, which meant they were the two most important variables for the distribution of *S. alterniflora*. The sum of *S_T_* arrived at 1.6994, which was three times than that of *S_F_*(0.5096). It indicated that there were strong interactions among variables. The *S_F_* of soil electrical conductivity (Tece) and soil drainage class (Drainage) ranked higher than their *S_T_*, while the *S_F_* of Soil pH (Tph) ranked lower than their *S_T_*, indicating interactions reduced the importance of Tece and drainage, while enhanced the importance of Tph. Meanwhile, some variables (Bio02, Elevation, Toc, Bio05, and Bio08) had great differences between *S_F_* and *S_T_*, especially for Bio02 (0.3663) and Elevation (0.2383), which meant that they had strong interactions with the others.

**Table 1:** Relative importance of different variables based on First‐order and total sensitivity index from global sensitivity analysis on the northern Chinese coast

Variables	First‐order sensitivity indices (*S_F_*)	Rank	Total sensitivity indices (*S_T_*)	Rank	Difference between first‐order and total sensitivity indices	Rank
Bio02	0.2775 (0.0552)	1	0.6437 (0.0818)	1	0.3663	1
Bio03	0 (0)	13	0.0212 (0.0061)	13	0.0211	13
Bio05	0.0338 (0.0185)	4	0.1531 (0.0637)	4	0.1193	4
Bio08	0.0333 (0.0205)	5	0.1267 (0.0511)	5	0.0935	5
Bio14	0.0001 (0.0001)	10	0.0257 (0.0065)	9	0.0256	9
Bio15	0 (0)	13	0.0149 (0.0027)	15	0.0148	15
Bio19	0.0001 (0.0001)	10	0.0229 (0.0080)	12	0.0229	11
Tece	0.0014 (0.0019)	6	0.0237 (0.0098)	11	0.0223	12
Tgravel	0.0001 (0.0001)	10	0.0268 (0.0084)	8	0.0267	8
Toc	0.0478 (0.0363)	3	0.1832 (0.1215)	3	0.1354	3
Tph	0 (0)	13	0.0277 (0.0080)	7	0.0277	7
Tsand	0.0002 (0.0001)	9	0.024 (0.0069)	10	0.0239	10
Drainage	0.0006 (0.0003)	7	0.0168 (0.0039)	14	0.0162	14
Tclass	0.0006 (0.0005)	7	0.0366 (0.0093)	6	0.0359	6
Elevation	0.1141 (0.0375)	2	0.3524 (0.0759)	2	0.2383	2
Sum	0.5096	‐	1.6994	‐	1.1899	‐

As seen from the Table 2, on the southern Chinese coast, whether the *S_F_* or *S_T_*, Elevation was by far the most important for the distribution of *S. alterniflora* with the values being 0.8561 and 0.9294, respectively, which were much greater than those of the second most important Bio02 with *S_F_* and *S_T_* being only 0.0658 and 0.1423, respectively. The *S_F_* of Bio14, Bio19, Tgravel, Tph, Tsand, and Tclass were 0, while the *S_T_* of them became non‐zero values. Moreover, the sum of *S_F_* is 0.9289 and the sum of *S_T_* was 1.3310. It indicated that there were still interactions among variables, but much weaker than that on the northern Chinese coast. Although the *S_F_* and *S_T_* of Elevation were much higher than Bio02, the difference was as great as that of Bio02, which indicated that both of them had distinguished interactions, especially for Bio02.

**Table 2:** Relative importance of different variables based on First‐order and total sensitivity index from global sensitivity analysis on the southern Chinese coast

Variables	First‐order sensitivity indices (*S_F_*)	Rank	Total sensitivity indices (*S_T_*)	Rank	Difference between first‐order and total sensitivity indices	Rank
Bio02	0.0658 (0.0166)	2	0.1423 (0.0362)	2	0.0764	1
Bio03	0.0019 (0.0007)	4	0.0230 (0.0023)	4	0.0211	4
Bio05	0.0002 (0.0005)	8	0.0191 (0.0021)	9	0.0189	10
Bio08	0.0012 (0.0008)	5	0.0211 (0.0018)	5	0.0199	5
Bio14	0 (0)	9	0.0188 (0.0014)	11	0.0187	12
Bio15	0.0029 (0.0017)	3	0.0250 (0.0043)	3	0.0221	3
Bio19	0 (0)	9	0.0185 (0.0017)	14	0.0185	14
Tece	0.0004 (0.0002)	6	0.0202 (0.0013)	6	0.0198	6
Tgravel	0 (0)	9	0.0191 (0.0015)	9	0.0190	8
Toc	0.0004 (0.0014)	6	0.0194 (0.0030)	7	0.0190	9
Tph	0 (0)	9	0.0187 (0.0015)	13	0.0187	13
Tsand	0 (0)	9	0.0183 (0.0017)	15	0.0183	15
Drainage	0 (0)	9	0.0188 (0.0015)	11	0.0188	11
Tclass	0 (0)	9	0.0193 (0.0016)	8	0.0192	7
Elevation	0.8561 (0.0380)	1	0.9294 (0.0156)	1	0.0733	2
Sum	0.9289	‐	1.3310	‐	0.4017	‐

### 3.3 Pure, shared, and coupling effects of climate change and sea level rise on the distribution of *S. alterniflora*

As shown in Table3, on the northern Chinese coast, the habitats of *S. alterniflora* increased by 30,934 km^2^, while decreased by 9,628 km^2^. 87.41% of habitats increment can be interpreted as being caused by pure effects of climate change, while only 0.53% by pure effects of sea level rise, 3.47% by shared effects, and 8.60% by coupling effects. For habitats reduction, the proportion caused by pure effects of climate change, pure effects of sea level rise, shared effects, and coupling effects accounted for 95.23%, 0.36%, 2.49%, and 1.91%, respectively. Pure effects of climate change mainly explained habitats change, while pure effects of sea level rise were quite low for whether habitats increment or reduction. Shared and coupling effects could explain 12.07% of habitats increment and only 4.4% of habitats reduction, which meant that the increased habitats were more deeply affected by interactions than the decreased habitats.

**Table 3:** Changed habitat caused by pure, shared, and coupling effects of climate changes and sea level rise on the northern Chinese

Northern Chinese coast	*H_p_clc_*	percentage	*H_p_slr_*	percentage	*H_s_ics_*	percentage	*H_c_ics_*	percentage	*H*_changed_	percentage
Increased habitats(km^2^)	27,038	87.41	164	0.53	1,073	3.47	2,659	8.60	30,934	100
Decreased habitats(km^2^)	9,169	95.23	35	0.36	240	2.49	184	1.91	9,628	100

Note

*H_p_clc_*: the changed habitats caused by pure effects of climate change; *H_p_slr_*: the changed habitats caused by pure effects of sea level rise; *H_s_ics_*: the changed habitats caused by shared effects from interactions of climate change and sea level rise; *H_c_ics_*: the changed habitats caused by coupling effects from interactions of climate change and sea level rise; *H*_changed_: the changed habitats.

As shown in Table 4, on the southern Chinese coast, the habitats of *S. alterniflora* increased by 69,417 km^2^, while decreased by 4,580 km^2^. It indicated that habitats will greatly increase in the future. 78.86% of habitats increment could be interpreted as being caused by pure effects of climate change, 1.25% by pure effects of sea level rise, 0.99% and 18.90% by shared and coupling effects, respectively. For the decrease habitats, the proportion caused by pure effects of climate change, pure effects of sea level rise, and shared effects accounted for 38.38%, 57.16%, and 4.45%, respectively. Coupling effects of interactions had no impacts on habitat reduction. Different from the northern coast, pure effects of climate change mainly explained habitats increment, while habitats reduction were explained by pure effects of sea level rise. For habitats increment, coupling effects were much greater than shared effects with the sum of shared and coupling effects of 19.89%, while shared effects were greater than coupling effects with the sum of only 4.45% for habitats reduction. The interactive way was different, which mainly appeared as coupling effects in habitats increment and shared effects in habitats reduction.

**Table 4:** Changed habitat caused by pure, shared and coupling effects of climate changes and sea level rise on the southern Chinese

Southern Chinese coast	*H_p_clc_*	Percentage	*H_p_slr_*	Percentage	*H_s_ics_*	Percentage	*H_c_ics_*	Percentage	*H*_changed_	Percentage
Increased habitats (km^2^)	54,743	78.86	868	1.25	685	0.99	13,121	18.90	69,417	100
Decreased habitats (km^2^)	1,758	38.38	2,618	57.16	204	4.45	0	0.00	4,580	100

Note

*H_p_clc_*: the changed habitats caused by pure effects of climate change; *H_p_slr_*: the changed habitats caused by pure effects of sea level rise; *H_s_ics_*: the changed habitats caused by shared effects from interactions of climate change and sea level rise; *H_c_ics_*: the changed habitats caused by coupling effects from interactions of climate change and sea level rise; *H*_changed_: the changed habitats.

### 3.4 Spatial distribution of changed habitat of *S. alterniflora* caused by pure, shared, and coupling effects of climate change and sea level rise on different regions

**Figure 5:**
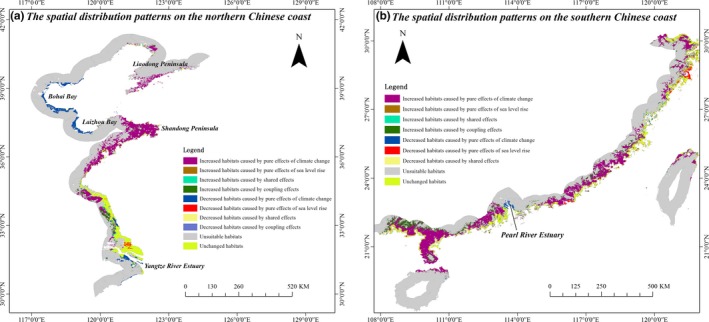
The spatial distribution of the changed habitats of *spartina alterniflora* caused by pure, shared, and coupling effects of sea level rise and climate change. (a) the northern Chinese coast (b) the southern Chinese coast

Along the northern Chinese coast (Figure 5a), the decreased habitats caused by pure effects of climate change mainly distributed on the Bohai bay, Laizhou bay, and Yangtze River Estuary, while the increased habitats mainly distributed on the Shandong Peninsula, Liaodong Peninsula, and northern Jiangsu Province. The changed habitats caused by shared and coupling effects were small and it mainly distributed in the coastal zone of Jiangsu province. On the southern Chinese coast (Figure 5b), the increased habitats caused by pure effects of climate change almost occupied the entire coast from Zhejiang to Guangxi Province. We found the increased habitats caused by coupling effects mainly distributed in the landward side of Guangxi and Guangdong and by shared effects were very small. Moreover, the decreased habitats caused by pure effects of sea level rise and climate change, which were very small, mainly distributed in Zhejiang coast and Pearl River Estuary, respectively.

## 4 DISCUSSION

Earlier studies used the rules provided by MaxEnt (percent contribution, permutation importance, and jackknife test) to determine the variable importance(Fand et al., 2014; Liu et al., 2018; Saatchi et al., 2008; Smart et al., 2012; Yao et al., 2016). Given interactions among variables were unavoidable, variable importance should be interpreted with caution when using these traditional methods (Parisien & Moritz, 2009). However, the global sensitivity analysis can reveal the importance of the main and total effects of different variables with considering interactions among variables (Haaker & Verheijen, 2004; Liu et al.,^2019^). Our research demonstrated that Bio02 and Elevation were the most important variables in controlling the distribution of *S. alterniflora* on the northern coast, whether main or total effects. The finding was consistent with previous studies (Kirwan et al., 2010; Liu, 2018; Priest, 2011). However, Elevation is by far the most important variable on the southern coast due to the width of tidal flat was narrow and the altitude has a relatively high limitation for the expansion of *S. alterniflora *(Gao et al., 2014). Bio02 and Elevation showed strong interactions among variables. An explanation for this might be that altitude can directly affect climatic factors such as temperature and precipitation and in turn climatic factors affect species distribution at different altitudes (Crosby et al., 2017; Idaszkin & Bortolus, 2011; Marangoni & Costa, 2012; Zhao et al., 2015). There were obvious interactions among variables influence the distribution of *S. alterniflora* on the Chinese coast, and interactions of the northern coast were much greater than that of the southern coast. Interactions reduced the importance of Tece and Drainage, while enhanced the importance of Tph on the northern Chinese coast. On the southern Chinese coast, interactions were low and it still enhanced the importance of Bio02, making the importance of Bio14, Bio19, Tgravel, Tph, Tsand, and Tclass change from zero to non‐zero.

It is recognized that climate change and sea level rise affect the species’ habitats (Cazenave & Cozannet, 2014; Garner et al., 2015; Reyer et al., 2013; Rincón, 2008; Valle et al., 2014). A number of researchers have reported that future climate change may further exacerbate invasive species expansion (Clout & Williams,^2009^; Crowl, Crist, Parmenter, Belovsky, & Lugo, 2008; Pyke et al., 2010). However, our results showed that habitats change was mainly caused by the pure effects of climate change. Climate change could not only exacerbate the expansion of *S. alterniflora*, but also cause its habitats reduction, especially on the northern Chinese coast. Pure effects of sea level rise were by far lower than that of climate change. The results differ from Hester's (2016) found that sea level rise will be dominant drivers in structuring *S. alterniflora* coastal wetlands and *S. alterniflora* is very sensitive to it. This discrepancy could be attributed to their research did not distinguish pure effects of sea level rise and interactions with other factors, and thus may overestimate the impact of sea level rise.

Our studies also showed that habitats change was influenced not only by pure effects of climate change and sea level rise but also by shared and coupling effects of their interactions, which is similar with the previous studies that habitats change was influenced by their interactions in a complex manner (Hering et al., 2009; Milad et al., 2011; Reyer et al., 2013; Wu, 2017). There were two ways of interactions (shared and coupling effects) between climate change and sea level rise. The shared and coupling effects mainly affected the habitats increment of *S. alterniflora *on the northern Chinese coast and independently affected habitats increment (coupling effects) and reduction (shared effects) on the southern coast. So, if interactions were ignored, the influences of climate change and sea level rise may be underestimated (Hering et al., 2009).

Most previous studies focused on habitats change (Cazenave & Cozannet, 2014; Garner et al., 2015; Reyer et al., 2013; Rincón, 2008; Valle et al., 2014), while almost no study has identified the spatial distribution patterns of habitats change caused by pure, shared, and coupling effects of climate change and sea level rise. Our results showed that habitats in Shandong Peninsula and Liaodong Peninsula will increase due to pure effects of climate change, while that of Bohai Bay, Laizhou Bay, and the Yangtze River Estuary will decrease. Therefore, we should pay attention to the distribution of *S. alterniflora* in Shandong Peninsula and Liaodong Bay to avoid further expansion. The changed habitats caused by shared and coupling effects mainly distributed in the landward side. It is because sea level rise will cause species, especially invasive species, to migrate to the landward side (Kebede & Mokrech, 2012; Kerstin et al., 2013; Ober & Martin, 2018), and thus caused interactions.

Overall, our findings illustrated that the distribution of *S. alterniflora* was controlled not only by the pure effects of climate changes and sea level rise, but also by the shared and coupling effects caused by their interactions in different regions. Thus, climate changes, sea level rise, and their interactions should be taken into consideration for robust predictions of the spatial distribution patterns of *S. alterniflora*. It will provide more scientific and reasonable suggestions for preventing and controlling the invasion of *S. alterniflora*.

Although ecological niche modeling (MaxEnt) is a superior technology for modeling the potential distribution of species, it has several limitations including its uncertainty and transferability (Phillips et al., 2006; Swanson et al., 2013). Given the model's uncertainty, our research was built on 10‐fold cross‐validation and multiple threshold rules, together with its high accuracy, and all of them supported the reliability of the results obtained (Elith & Yates, 2015; Radosavljevic et al., 2013). MaxEnt assumes that species will not exhibit phenotypic adaptation to new environmental conditions (Hernandez et al., 2006). Our model did not account for species dispersal, while the seeds of *S. alterniflora* can spread over long distances by wind and waves. Thus, further work is needed to combine the dispersal of *S. alterniflora* to better predict its actual distribution. Furthermore, we only assumed that the average sea level will rise by 1 meter without considering the spatial heterogeneity of sea level rise. Abiotic environmental factors such as interspecies competition and ecosystem dynamics could also influence *S. alterniflora*'s survival and colonization success (Woolfolk, Wasson, 2013; Garner et al., 2015). Therefore, further studies require considering the effects of biological factors such as species dispersal, competition, the spatial patterns of sea level rise in different regions.

## AUTHOR CONTRIBUTIONS

Haibo Gong formed the original idea and wrote the original manuscript; Huiyu Liu offered valuable comments and was responsible for the manuscript revisions; FuSheng Jiao created figures and tables; Zhenshan Lin and Xiaojuan Xu analyzed the data.

## Data Availability

The datasets are available at http://data.gbif.org/, www.worldclim.com, http://webarchive.iiasa.ac.at/Research/LUC/External-World-soil-database/HTML/, and https://www.cresis.ku.edu/content/research/maps.
