# Supplementary material for: Pure, shared, and coupling effects of climate change and sea level rise on the future distribution of Spartina alterniflora along the Chinese coast
Source: Ecol Evol. 2019 Apr 16;9(9):5380–91. doi: 10.1002/ece3.5129 (PMC6509381; doi:10.1002/ece3.5129)
Supplement: Supplementary file 1 [file ECE3-9-5380-s001.docx]

**Ecology and Evolution**

SUPPORTING INFORMATION

**Pure, shared, and coupling effects of climate change and sea level rise on the future distribution of *Spartina alterniflora* along the Chinese coast**

Haibo Gong, Huiyu Liu*, Fusheng Jiao, Zhenshan Lin, Xiaojuan Xu

**Appendix Figure S1** Spearman rank correlation analysis of 19 bioclimatic variables (*p < .05. **p < .01. ***p < .001)

**Appendix Figure S2** The relative importance of 19 bioclimatic variables based on feature selection using Burota algorithm

**Appendix Table S1** Variables used in ecology niche model and probability distribution functions of variables on the northern Chinese coast

| Variable types | Variables | Description | Distribution | Expression | Square Error | Chi Square Test |
| --- | --- | --- | --- | --- | --- | --- |
| Climate | Bio02 | Mean diurnal range (Mean of monthly | Beta | 13 * BETA(13, 4.82)-0.001 | 0.0018 | p-value<0.005 |
| Climate | Bio03 | Isothermality | Beta | 17 * BETA(9.74, 4)+13 | 0.0026 | p-value<0.005 |
| Climate | Bio05 | Max temperature of warmest month | Beta | 34 * BETA(37.4, 4.56)-0.001 | 0.0099 | p-value<0.005 |
| Climate | Bio08 | Mean Temperature of Wettest Quarter | Normal | NORM(24.2, 1.43) | 0.0138 | p-value<0.005 |
| Climate | Bio14 | Precipitation of Driest Month | Gamma | GAMM(9.46, 1.38)+0.5 | 0.0147 | p-value<0.005 |
| Climate | Bio15 | Precipitation Seasonality | Beta | 105 * BETA(1.87, 1.52)+15 | 0.0187 | p-value<0.005 |
| Climate | Bio19 | Precipitation of Coldest Quarter | Gamma | GAMM(43.8, 0.981)+6 | 0.0146 | p-value<0.005 |
| Soil | Tece | Soil electrical conductivity | Weibull | WEIB(0.886, 6.26) | 0.0157 | p-value<0.005 |
| Soil | Tgravel | Volume percentage gravel | Gamma | GAMM(3.9, 2.05)+0.5 | 0.1220 | p-value<0.005 |
| Soil | Toc | Soil organic carbon | Beta | 4*(1.71,7.25) | 0.0001 | p-value<0.005 |
| Soil | Tph | Soil pH | Beta | 5*(2.98,1.73)+3.5 | 0.0490 | p-value<0.005 |
| Soil | Tsand | Percentage sand | Gamma | GAMM(7.64,4.06)+9.5 | 0.0777 | p-value<0.005 |
| Soil | Drainage | Soil drainage class | Discrete | - | - | p-value<0.005 |
| Soil | Tclass | Soil unit symbol (FAO-90) | Discrete | - | - | p-value<0.005 |
| Topography | Elevation | Elevation | Beta | 1290* BETA(0.395, 7.65)-9 | 0.0041 | p-value<0.005 |

**Appendix Table S2** Variables used in ecology niche modelling and probability distribution functions of variables on the southern Chinese coast

| Variable types | Variables | Description | Distribution | Expression | Square Error | Chi Square Test |
| --- | --- | --- | --- | --- | --- | --- |
| Climate | Bio02 | Mean diurnal range (Mean of monthly | Normal | NORM(7.16, 0.839) | 0.0019 | p-value<0.005 |
| Climate | Bio03 | Isothermality | Beta | 36 * BETA(4.16, 4.58)+16 | 0.0046 | p-value<0.005 |
| Climate | Bio05 | Max temperature of warmest month | Weibull | WEIB(18.1, 19.2)+14 | 0.0204 | p-value<0.005 |
| Climate | Bio08 | Mean Temperature of Wettest Quarter | Beta | 23 * BETA(6.53, 1.83)+7 | 0.0272 | p-value<0.005 |
| Climate | Bio14 | Precipitation of Driest Month | Gamma | GAMM(9.87, 2.71)+8 | 0.0245 | p-value<0.005 |
| Climate | Bio15 | Precipitation Seasonality | Beta | 97 * BETA(5.38, 5.78)+19 | 0.0128 | p-value<0.005 |
| Climate | Bio19 | Precipitation of Coldest Quarter | Gamma | GAMM(36.7, 3.14)+33 | 0.0173 | p-value<0.005 |
| Soil | Tece | Soil electrical conductivity | Beta | 9 * BETA(2.9, 45.3) | 0.0098 | p-value<0.005 |
| Soil | Tgravel | Volume percentage gravel | Gamma | GAMM(8.18, 1.42) | 0.1392 | p-value<0.005 |
| Soil | Toc | Soil organic carbon | Beta | 7 * BETA(4.6, 17.9) | 0.0217 | p-value<0.005 |
| Soil | Tph | Soil pH | Beta | 5 * BETA(0.906, 1.7)+3.5 | 0.0389 | p-value<0.005 |
| Soil | Tsand | Percentage sand | Gamma | GAMM(5.57, 6.59)+3.5 | 0.0765 | p-value<0.005 |
| Soil | Drainage | Soil drainage class | Discrete | - | - | p-value<0.005 |
| Soil | Tclass | Soil unit symbol (FAO-90) | Discrete | - | - | p-value<0.005 |
| Topography | Elevation | Elevation | Beta | 3730*BETA(0.219,3.73)-22 | 0.0022 | p-value<0.005 |
